# The Impact of Stroke Subtype on Recovery and Functional Outcome after Inpatient Rehabilitation: A Retrospective Analysis of Factors

**DOI:** 10.3390/life12091295

**Published:** 2022-08-23

**Authors:** Rathi Ratha Krishnan, Edgar Quan Yi Yeo, Chien Joo Lim, Karen Sui Geok Chua

**Affiliations:** 1 Centre of Rehabilitation Excellence (CORE), Tan Tock Seng Hospital Rehabilitation Centre, Singapore 569766, Singapore; 2 Lee Kong Chian School of Medicine, Nanyang Technological University, Singapore 308232, Singapore; 3 Yong Loo Lin School of Medicine, National University of Singapore, Singapore 117597, Singapore; 4 MOH Holdings, Singapore 099253, Singapore; 5 Department of Orthopaedic Surgery, Woodlands Health, Singapore 768024, Singapore

**Keywords:** cerebral infarction, cerebral hemorrhage, rehabilitation, stroke, functional outcome

## Abstract

The aims of this study were to compare inpatient rehabilitation outcomes between acute stroke subtypes of Cerebral Infarction (CI) and Intracerebral Hemorrhage (ICH), and to determine the predictors of discharge outcomes. A retrospective study of stroke inpatients was carried out using the discharge Functional Independence Measure (FIM) as the primary outcome measure. Relationships between stroke subtype, rehabilitation impairments, and medical complications on FIM -gain were analyzed. Altogether, 280 datasets including 211 (75.4%) CI and 69 (24.6%) ICH were analyzed. ICH patients were significantly younger than CI patients (55 years ICH vs. 64.0 years CI years, *p* < 0.001), had a 10-fold higher proportion needing ICU admission (ICH 82.6% vs. CI 7.6%, *p* < 0.001), and had significantly lower total admission FIM scores (67 points ICH vs. 74 CI points, *p* = 0.006), with lower motor-FIM scores in particular (38 points ICH vs. 48 points CI, *p* = 0.003). Significant functional improvements after inpatient rehabilitation, i.e., FIM gain, occurred regardless of stroke subtype (FIM-ICH Δ 27 vs. FIM-CI Δ 21, *p* = 0.05). Despite significantly worse initial stroke severity, ICH patients achieved similar functional gains, independence levels, and return-home rates compared with their CI counterparts after inpatient rehabilitation.

## 1. Introduction

Stroke is a disorder characterized by significant impairment of sensorimotor and cognitive functions. Stroke is a leading cause of death and disability globally, i.e., The Global Burden of Disease Study 2019 placed stroke as one of the five leading causes of morbidity. Despite best stroke management and access to rehabilitation, 65% of survivors live with long-term disabilities and the economic burden of stroke continues to increase with global population aging [1]. Stroke is also the fourth leading cause of death in Singapore, accounting for 17% of all deaths [2]. It is also one of the top 10 causes of hospitalization [3], with ~40% of survivors experiencing limitations in functional status [4].

Interdisciplinary combined acute and rehabilitation stroke units have been shown to reduce combined death and/or dependency, need for institutionalization, and hospital length of stay. Having specialized subacute stroke rehabilitation was shown to reduce mortality in comparison to general medical management [5]. A critical review of rehabilitation interventions by Cifu et al. revealed that early initiation of rehabilitation services had a strong positive relationship with functional outcome after stroke, while the use of specialized types of therapy services and greater therapy intensity also improved functional outcome at discharge and follow-up [6].

The functional outcome of stroke survivor subtypes is of interest and the results in the literature are mixed. Studies conducted on unselected stroke patients reported patients with ICH as having either poorer [7] or similar functional outcomes as CI patients [8,9,10]. In a study of rehabilitation patients by Paolucci et al., ICH patients fared better [11]. This study involved a case-control design of 270 stroke rehabilitation patients (135 CI, 135 ICH patients). The Canadian Neurological Scale (CNS) and Rivermead Mobility Index (RMI) were the main outcome measures administered on arrival and discharge. CI and ICH patients were matched by basal stroke severity (CNS score), basal disability (Barthel index score), age, gender, and onset-admission interval. At discharge, both CI and ICH stroke groups made significant gains in CNS, BI, and RMI, but the ICH patients were found to have significantly higher CNS and RMI on discharge.

However, other studies found no significant difference in functional outcome between the two stroke subtypes [12,13,14]. Kelly et al. conducted a retrospective study on 1064 stroke rehabilitation patients (*n* = 871 CI patients and *n* = 193 ICH patients), and used the Functional Independence Measure (FIM) score as the outcome measurement [13]. It was found that, on admission, ICH patients had significantly lower FIM scores and cognitive FIM scores compared to CI patients, but upon discharge from rehabilitation, there was no difference in discharge FIM score. This was because ICH patients had significantly greater changes in total, motor, and cognitive FIM score compared to CI patients. FIM efficiency was not found to be significantly different between the two stroke groups.

Despite the higher ICH incidence in Asia compared to Caucasian data (15.4% vs. 10–15% [3,15,16], comparative studies with regard to stroke subtype and rehabilitation outcome in Asia are scarce. A local prospective functional outcome study by Ng YS et al. involving 1332 stroke patients, which took place a decade ago, found that ICH patients presented with a lower admission FIM score but a higher average FIM gain score upon discharge from inpatient rehabilitation [17]. They found no significant difference in outcomes between the two stroke types. A retrospective study in Taiwan reported subcortical ICH patients having greater functional improvement and greater late-phase recovery than their CI counterparts following their rehabilitation program, even though their stroke was significantly more severe on admission [18].

Therefore, the objectives of the current study were (i) to compare the functional outcomes on admission and discharge from inpatient rehabilitation between ICH and CI patients and (ii) to determine if there are significant demographic, clinical, or stroke-related predictors of rehabilitation discharge using FIM.

## 2. Materials and Methods

### 2.1. Study Design

A retrospective study of anonymized hospital Electronic Medical Records (EMRs) for consecutive patients who underwent inpatient stroke rehabilitation at a single rehabilitation facility was conducted from 1 January 2016 to 31 December 2017. Ethics approval was granted by the National Healthcare Group (NHG) institutional review boards (NHG-DSRB 2018/00228). Informed consent was waived by NHG-DSRB as no identifiers were involved.

### 2.2. Study Setting

The study was conducted at the Tan Tock Seng Hospital Rehabilitation Centre, Singapore, which receives and admits patients from acute stroke units after neurological stabilization through a fast-track day 2 physician and virtual screening daily work-week pathway. Patients are then transferred to the rehabilitation center, which is located 12 km away from the acute hospital stroke unit. The inpatient rehabilitation program, led by physiatrists, consists of rehabilitation therapies (supervised 3 h daily, 5.5 days a week), delivered by an interdisciplinary team of physiotherapists, occupational therapists, speech pathologists, nurses, social workers, and psychologists. The therapist-to-patient ratio of 1:8 and inpatient rehabilitation program are modelled following the neurodevelopmental techniques and Bobath principles, with daily medical consultation. Mental health and mood screening for post-stroke depression and anxiety are provided by psychiatrists and psychologists, and social interventions are provided by care counsellors. Paramedical consultations with nutritionists, pharmacists, orthotists, and prosthetists are provided onsite on a weekly basis. Patients are also evaluated for suitability for upper- and lower-limb rehabilitation robot-aided therapies, (e.g., Lokomat^®^ robotic-driven orthosis, Armeo^®^Power and Armeo^®^Spring upper-limb robots [Hocoma, AG, Volketswil, Switzerland, www.hocoma.com, (accessed on 14 August 2022)], supervised treadmill training, virtual reality commercial gaming platforms, neuromuscular electrical stimulation, and EMG biofeedback devices. Weekly multidisciplinary rehabilitation staff conferences are conducted to set and review rehabilitation goals, track functional progress, and discharge planning. 

The Functional Independence Measure (FIM)—the main outcome measure to measure change in function during inpatient rehabilitation—is routinely recorded within 72 h of admission and discharge by the rehabilitation staff, all of whom are trained and accredited in the use of the FIM. Data are subsequently entered into a prospective functional database registry (National Healthcare Group Standing Database Registry, NHG SDB 2010/0039). 

### 2.3. Study Subjects

Subjects’ EMRs were enrolled based on the following inclusion criteria: (i) age 18 to 85 years; (ii) first clinical stroke; (iii) diagnosis of stroke made by a neurologists or neurosurgeons, confirmed by neuroimaging (CT, MRI, cerebral angiography); (iv) admitted directly from acute hospitals; and (v) within 180 days of stroke. 

Subjects’ EMRs were excluded if they had any of the following exclusion criteria: (i) strokes secondary to Spontaneous Subarachnoid Hemorrhage (SAH), brain trauma, or infections; (ii) nonrehabilitation reasons for admission; (iii) failure to complete inpatient rehabilitation; and (iv) incomplete FIM data. 

### 2.4. Study Outcomes

A case record form was derived from the following extracted variables based on their EMR for eligible case records: (i) demographic variables; (ii) acute stroke characteristics, admission to, and length of intensive care unit (ICU) stay and acute hospital length of stay (LOS-A/days); (iii) presence/absence of rehabilitation impairments on admission. Initial stroke severity upon admission to acute stroke units was graded using the National Institutes for Health Stroke Scale (NIHSS) for CI and the Glasgow Coma Scale (GCS) for ICH patients, respectively. 

Upon admission to the rehabilitation unit, the presence of cognitive impairment was determined based on patients’ Montreal Orientation and Cognitive Assessment (MOCA) score as <24/30 within 72 h of rehabilitation admission [13]. Motor impairments were quantified using the Trunk Impairment Scale (TIS) [19] and the Fugl-Meyer Motor Assessment Scale (FMA) [20] for the upper and lower limbs, respectively.

The rehabilitation length of stay (LOS-R/days), discharge disposition, and numbers of intrarehabilitation complications, defined as those which disrupted rehabilitation or needed treatment, were recorded. 

For this study, the primary outcome measure was functional outcome upon discharge from rehabilitation, as measured by the total discharge FIM (Td-FIM). The FIM is an 18-item instrument that is widely used in the rehabilitation population to measure a patient’s ability to perform Activities of Daily Living (ADLs) across 6 areas (self-care, sphincter control, transfers, locomotion, communication, and social cognition) (https://www.udsmr.org/products/international, accessed on 14 August 2022). Total admission FIM (Ta-FIM) and total discharge FIM (Td-FIM) scores (18–126), motor FIM (m-FIM) subscores (13–91), and cognitive FIM (c-FIM) subscores (5–35) were tabulated. In addition, mean FIM gain (FIM-G) and mean FIM efficiency (FIM-E) were derived as follows: (i) FIM-G = [Td-FIM score–Ta- FIM score] and (ii) FIM-E = [FIM-G]/[LOS-R (days)]. All individual values were aggregated.

The STROBE guidelines were used to strengthen the reporting of this study (https://www.equator-network.org/reporting-guidelines/strobe/, accessed on 14 August 2022).

### 2.5. Statistical Analysis

Statistical analyses were carried out using IBM Statistical Product and Service Solutions (IBM SPSS Statistics for Windows, version 22, IBM Corp., The Armonk, NY, USA) and STATA version 14.0 (StataCorp LP, College Station, TX, USA). Descriptive statistics were used to summarize sociodemographic and clinical data. Data were presented as either mean and standard deviation or median and interquartile range where appropriate. Data were assessed for normality assumptions using the skewness, kurtosis, and histogram. The differences in sociodemographic characteristics and admission and rehabilitation characteristics between CI and ICH subtypes were explored using independent sample *t* test, Mann–Whitney U test, Pearson chi-squared test, and Fisher exact test. Regression modelling analyses were carried out to examine predictors of functional outcome as measured by Td-FIM. Simple linear regression analysis was used to explore the factors impacting total discharge FIM, and the variables were included into the variable selection process in the multiple linear regression model. A stepwise variable selection method was used, and assumption of heteroscedasticity of the final model was tested using Breusch–Pagan/Cook–Weisberg test. The level of significance was set at *p* < 0.05 for all tests.

## 3. Results

### 3.1. Patient Recruitment Flow

Of 363 consecutive rehabilitation EMRs identified from inpatient admissions during the study period, 83 EMRs were excluded for the following reasons: 68 had prior strokes, 9 had SAH, 5 failed to complete rehabilitation, and 1 was admitted >180 days after being diagnosed with a stroke. In total, 280 datasets were available for analysis, including 211 (75.4%) CI and 69 (24.6%) ICH patients. 

### 3.2. Patient Demographics and Acute Stroke Characteristics

The demographic and acute stroke characteristics of the cohort and CI/ICH subgroups are presented in Table 1. The mean (SD) age of the 280 patients was 61.8 (12.38) years, 43.6% (122/280) of the sample were aged > 65 years, 40.4% (113/280) were females, and 82.1% (230/280) were ethnic Chinese. Gender, handedness, and stroke laterality were similar between the CI and ICH stroke subtypes. 

ICH survivors were significantly younger than CI patients by an average of 8.8 years (ICH mean 55.2 years vs. CI mean 64.0 years, *p* < 0.001). A significantly higher proportion of ICH patients were of Chinese ethnicity compared to CI patients (ICH 92.8% vs. CI 78.7%, *p* = 0.010), and significantly more ICH compared to CI patients were employed at stroke diagnosis (ICH 71% vs. CI 53.1%, *p* = 0.009).

ICH patients had a significantly longer median LOS-A by 6 days compared with CI patients (ICH median 15 days vs. CI median 9 days, *p* < 0.001). Furthermore, ICU admission rates were 10 times higher in ICH patients compared with CI patients (ICH mean 57/69 (82.6%) vs. CI mean 16/211 (7.6%), *p* < 0.001), whilst the median duration of ICU stay of 72 h was similar for both stroke subtypes (*p* = 0.69). 

Comparing stroke severities, both groups were largely similar on respective acute clinical gradings: 6.2% (13/211) of CI patients had a severe stroke (NIHSS score of 21–42), while 7.2% (5/69) of ICH presented with severe stroke (GCS 3–8). 

### 3.3. Rehabilitation Impairments

The rehabilitation characteristics of the cohort and CI/ICH subgroups are presented in Table 2. 

On admission to the rehabilitation unit, in general, the ICH subtype had a high prevalence of multiple impairments, notably motor impairments (91%, 255/280), sensory impairments (40%, 112/280), post-stroke cognitive impairments (70%, 196/280), and dysphagia (50%, 140/280), which was severe in a minority who required NGT feeding (16.8%, 47/280). In this regard, the ICH subgroup also had a ~3-fold higher prevalence of severe dysphagia requiring NGT, (ICH 22/69 (31.9%) vs. CI 25/211 (11.8%), *p* < 0.001) compared to the CI subgroup, higher sensory impairment burden (ICH 55.1% (38/69) vs. CI 35.1% (74/211), *p* = 0.004), and weaker trunk impairment scale (TIS) scores (ICH median TIS 11.0 vs. CI median 14.0, *p* = 0.014). All five patients (7.2%) who underwent tracheostomy for prolonged ventilation belonged to the ICH subtype

With regard to the presence of aphasia and cognitive and motor impairments (the latter being measured by MOCA and FMA scores, respectively), the rehabilitation admission profiles for ICH vs. CI patients were similar. The median FMA scores were worse in ICH patients compared to the CI subtypes for both upper (CI 43 vs. ICH 26, *p* = 0.08) and lower limbs (CI 22 vs. ICH 14, *p* = 0.064), although this was not statistically significant.

### 3.4. Admission and Discharge Rehabilitation Functional Status

The admission and discharge functional outcomes are presented in Table 3, Table 4 and Table 5. The median (IQR) Td-FIM was 98 (41), representing a significant improvement from the median (IQR) Ta-FIM of 72 (39) (*p* < 0.001), which indicates that the average patient did not require physical aid after rehabilitation, as FIM ≥ 91 indicates at least a modified independence level. There was no significant difference in the proportion of patients with Td-FIM ≥ 91 between the stroke types (CI 60.7% vs. ICH 53.6%, *p* = 0.302). The majority of these significant functional gains were achieved in m-FIM [∆ 23] compared to c-FIM [∆ 4, *p* < 0.001]. The median FIM-E was 0.786 and the median FIM-EF (IQR) was 0.85 (0.73). 

ICH patients had a significantly lower median Ta-FIM (ICH 67 vs. CI 74, *p* < 0.006) and admission m-FIM (ICH 38 vs. CI 48, *p* < 0.003). However, FIM-gain for both groups was not significantly different, although the ICH groups tended to exhibit greater gains by +6 FIM points, compared to the CI groups (ΔFIM ICH 27 vs. ΔFIM CI 21, *p* = 0.05).

LOS-R was not significantly dissimilar between both groups, although ICH patients stayed longer by a median of 7 days (CI 27 vs. ICH 34, *p* = 0.09), while the overall return-home rate of 87.5% was similar for both groups. (Table 5). There were no significant differences in the presence of any complications during rehabilitation (CI 39.8% vs. ICH 37.7%, *p* = 0.778) or in the discharge FIM score (CI 99 vs. ICH 94, *p* = 0.259).

Multivariate regression analyses for variables impacting the total discharge FIM score are presented in Table 6. The adjusted R squared score was 75.9%. Variables that were significant in the final model included employment status (*p* < 0.001), stroke type (*p* = 0.012), acute LOS (*p* < 0.001), TIS (*p* < 0.001), motor FIM (*p* < 0.001), and cognitive FIM (*p* < 0.001). Age, gender, and acute impairments were not found to be significant. 

## 4. Discussion

Findings from this study confirmed that 4 weeks to 30 days of inpatient rehabilitation conferred objective functional benefits for the entire sample, with a significant FIM-G (22.0), which met the Minimal Clinically Significant Difference (MCID) thresholds for the total FIM-D, FIM-M (23.0), and FIM-C (4.0) subscores at 22, 17, and 3, respectively [21]. The FIM-G difference of +6 between the ICH (higher) and CI groups was at borderline significance (*p* = 0.05) likely due to the relatively small sample size. ICH patients stayed on average a week longer than CI patients, although this was not statistically significant. This could be explained by a higher number of days needed by ICH patients to achieve their goals as FIM-E per day gain was similar for both subtypes. 

ICH patients were admitted to the inpatient rehabilitation unit with a significantly lower median admission total FIM (FIM-A ICH 67 vs. FIM-A CI 74, *p* < 0.006); this difference is attributed to lower motor rather than cognitive FIM in the ICH groups (ICH 38 vs. CI 48, *p* = 0.003). Thus, ICH patients presented with a significantly poorer motor functional status on rehabilitation admission, which can be explained by greater lower-limb impairment in the ICH group, with a lower-limb median FMA of 14 compared to 22 for the CI group (14 being the cut-off for severity), as well as significantly lower TIS in the ICH compared to the CI group (TIS ICH 11.0 vs. TIS CI 14.0, *p* = 0.014). That more severe strokes are associated with poorer admission and discharge FIM scores is not surprising, as has been previously shown for both ischemic [22] and hemorrhagic stroke [16]. Cognitive FIM, TIS, and upper-limb FMA were similar between groups and are not likely to account for the difference.

Similar discharge FIM scores were achieved in both groups, (ICH FIM-D 94/126 vs. CI FIM-D 99/126, *p* = 0.259). Scores above 90 indicate modified independence levels without the need for a carer, implying mild impairment. There was no significant difference in the proportion of patients with Td-FIM ≥ 91 between the stroke types (CI 60.7% vs. ICH 53.6%, *p* = 0.302). Chu et al. postulated that participating in rehabilitation aids in reorganization of the neural network, increases neuroplasticity and angiogenesis, and augments hematoma and toxic blood product clearance in ICH patients [18]. It was also noted in a study by Salvadori et al. that better functional outcomes in ICH vs. CI patients were seen in studies conducted in specialized rehabilitation settings, while worse or equivocal outcomes were seen in studies conducted in community or acute hospital settings [23]. Ng et al. also reported that all patients, regardless of their stroke subtypes, made clinically significant functional gains on completion of inpatient rehabilitation [17]. 

The median FIM gain for ICH patients tended to be higher than CI patients (ICH 27 vs. CI 21, *p* = 0.05). Only the ICH group achieved FIM-G exceeding the MCID threshold for acute stroke Δ22, [23] compared to CI patients. Greater FIM gain in ICH patients was consistent with the local study by Ng et al. [17]. Possible reasons for these differences are as follows: (i) the resolution of toxic blood products, cerebral edema, and the mass effect may result in greater improvements in ICH patients, accounting for the greater FIM gain; (ii) a tendency to a longer rehabilitation LOS for ICH patients by 1 week (ICH median 34 days vs. CI median 27 days, *p* > 0.05) may have allowed more time for motor and functional goal attainments; (iii) a significantly younger ICH group with a higher employment status may attest to higher premorbid cognitive reserve and overall better outcomes. 

In terms of demographics, the study cohort consisted of 24.6% ICH survivors and this proportion of hemorrhagic strokes was similar to the data reported by Singapore’s stroke registry [2,24] and similar to the rest of Asia. Moreover, these data are in accordance with a significantly higher hemorrhagic stroke prevalence than that for Caucasian populations (ICH Asia 15–40% vs. ICH Caucasian 10–15%) [15,16]. 

In general, the overall mean age of patients in this sample was younger compared to the average age of general stroke patients in Singapore (mean age sample 62 years vs. mean age population 68 years) [24]. This finding concurs with that of Ng et al. in a study conducted 9 years ago in which the mean age was 64.1 years [17]. This may be due to the studies being conducted in rehabilitation units where patients are screened and selected for admission to the program. In particular, ICH patients in this study were also found to be significantly younger compared to their CI counterparts by ~9 years (mean age CI 64 years vs. mean age ICH 55 years, *p* < 0.001). This difference is largely similar to the age difference between ICH and CI patients in the general stroke population (~6 years) [24].

A higher proportion of ICH patients was employed (71%) compared to CI patients (53%), and this was likely related to their younger mean age, the local official retirement age being 62 years. 

In general, hemorrhagic strokes have more complex acute presentations and patients are more severely affected [25]. This may be due to the difference in the mechanisms of brain injury and recovery in patients with CI and ICH. In ICH, there is toxicity of lysed blood products on the brain parenchyma and vessels, mass effect from hematoma, in addition to ischemia, cerebral edema, and inflammation [26]. This was the probable explanation for the higher rates of ICU admission by 10-fold in the ICH subtype (ICH 82.6% vs. CI 7.6%, *p* < 0.001) and longer acute LOS (1 ICH 5.5 days vs. CI 9.5 days, *p* < 0.001) in the ICH compared to CI patients. 

The tendency towards a longer LOS could be explained in several ways: higher complexity of care needs in ICH patients as reflected by significantly higher incidences of NGT (ICH 31.9% vs. CI 11.8%, *p* < 0.001), tracheostomy (ICH 7.2% vs. CI 0%, *p* = 0.001), and sensory impairments (ICH 55.1% vs. CI 35.1, *p* = 0.004) compared to CI patients. Despite this, FIM efficiency was similar in both groups. This suggested that ICH patients made similar daily and overall improvements as CI patients, despite the worse initial severity, complexity, and longer acute hospital stay. 

Intrarehabilitation complication rates (40%CI vs. 38% ICH, *p* > 0.05) and return-home rates were similar (89%CI vs. 84% ICH, *p* > 0.05). The high return-home rate could be related to mild disability levels after rehabilitation and good familial and local societal supports. This finding concurs with that of Ng et al. in which 87.7% of patients were discharged home after rehabilitation. 

Regarding the analysis of predictive factors for FIM-D using multivariate regression modelling, premorbid employment status rather than age, admission FIM scores, and stroke subtype (i.e., ICH) were significantly associated with higher discharge FIM scores. This is congruent with the known literature, which shows that both premorbid employment status and younger age are important predictors of better outcome as they suggest higher cognitive and physical reserves [27]. Patients who were employed before the stroke occurrence were likely to be younger, with a higher functional status and increased cognitive or social resilience, which may account for the better outcome associated with employment. 

Older patients often experience multiple comorbid medical complications and may also face age-related frailty resulting in lowered physical reserves and a premorbid decline in functional status. In comparison, the ICH stroke subtype contributed negatively to FIM gain by ~5 points, while being employed prior to stroke added +6.0 FIM points. The regression model also suggested that the presence of rehabilitation complications, such as UTI or pneumonia, did not lead to worse functional (FIM) outcomes. This could possibly be related to appropriate expedient medical management and team efforts to prevent pulmonary aspiration, removal of urinary catheters to establish physiological voiding, intensive targeted inpatient speech therapy when resuming supervised and modified oral feeding, and verticalization maneuvers to prevent atelectasis. Our model also found that both admission motor and cognitive FIM subscores and TIS scores were significant although weak predictors of discharge FIM.

### Study Limitations 

Our study had several limitations. Firstly, it is a retrospective, single center study, with a relatively small sample size and an under-representation of elderly patients (44% were aged >65 years) where stroke incidence is highest; thus, our findings may have limited generalizability to the general stroke population and may contain a selection bias as a result of the assessing physicians prior to entry to inpatient rehabilitation. Lastly, there is a lack of long-term outcomes postdischarge in terms of societal integration, quality of life, and healthcare cost data.

## 5. Conclusions

The findings from this study support the functional benefits of a month-long inpatient stroke rehabilitation program in reducing impairments, improving functional outcomes and independence, and returning patients home regardless of age or stroke subtype. Despite poorer admission status and higher acute complexity, our findings demonstrated that ICH patients achieved comparable FIM gains and functional independence without the need for a caregiver upon discharge as CI patients, without increased complication rates or significantly longer lengths of rehabilitation stays, which is in concurrence with similar Asian cohorts. Premorbid employment predicted better discharge function, while the ICH subtype and lower admission cognitive FIM negatively predicted discharge rehabilitation outcome.

We conclude that an overly negative attitude towards a poor functional prognosis for hemorrhagic stroke survivors with initial worse acute stroke scores, higher initial acute stroke complexity, and lower admission functional status after rehabilitation should be discouraged.

## Figures and Tables

**Table 1:** Comparison of demographic and acute stroke characteristics by stroke subtype.

Variable	Total (*n* = 280)	CI (*n* = 211)	ICH (*n* = 69)	*p*-Value
**Age**				
Age in years, mean (SD)	61.81 (12.38)	63.98 (11.95)	55.19 (11.34)	<0.001 ^a^
Age 65 or greater (%)	122 (43.6)	107 (50.7)	15 (21.7)	<0.001 ^c^
**Gender (%)**				
Male	167 (59.60)	125 (59.20)	42 (60.90)	0.811 ^c^
Female	113 (40.40)	86 (40.80)	27 (39.10)	
**Race (%)**				
Chinese	230 (82.1)	166 (78.7)	64 (92.8)	0.010 ^c^
Non-Chinese	50 (17.9)	45 (21.3)	5 (7.2)	
Malay	30 (10.7)	27 (12.8)	3 (4.3)	
Indian	12 (4.35)	12 (5.7)	0 (0)	
Others	8 (2.9)	6 (2.8)	2 (2.9)	
**Employment (%)**				
Yes	161 (57.5)	112 (53.1)	49 (71)	0.011 ^c^
No	119 (42.5)	99 (46.9)	20 (29)	
**Handed (%)**				
Right-handed	268 (95.7)	200 (9 4.8)	68 (98.6)	0.305 ^d^
Left-handed	12 (4.3)	11 (5.2)	1 (1.4)	
**Side of stroke (%)**				
Unilateral	268 (95.7)	202 (95.7)	66 (95.7)	>0.950 ^d^
Bilateral	12 (4.3)	9 (4.3)	3 (4.3)	
**Length of stay in acute hospital,** median (IQR)	10.0 (9)	9 (8)	15 (12)	<0.001 ^b^
**ICU stay (%)**				
Yes	73 (26.1)	16 (7.6)	57 (82.6)	<0.001 ^c^
No	207 (73.9)	195 (92.4)	12 (17.4)	
**Length of stay in ICU,** median (IQR)	3 (4)	3 (7)	3 (4)	0.690 ^b^
**Stroke Severity on initial admission**				
**NIHSS (%)**				
Mild (1–4)		90 (42.7)		
Moderate (5–15)		97 (46.0)		
Moderate-severe (16–20)		11 (5.2)		
Severe (21–42)		13 (6.2)		
**GCS (%)**				
Mild (13–15)			45 (65.2)	
Moderate (9–12)			19 (27.5)	
Severe (3–8)			5 (7.2)	
**Type of impairment on rehabilitation admission (%)**				
Tracheostomy	5 (1.8)	0 (0)	5 (7.2)	0.001 ^d^
Ventriculo-peritoneal shunt	1 (0.4)	1 (0.5)	0 (0)	>0.950 ^d^

^a^ Independent Sample t test; ^b^ Mann–Whitney U test; ^c^ Pearson Chi Square test; ^d^ Fisher Exact test.

**Table 2:** Comparison of admission rehabilitation characteristics by stroke subtype.

Variable	Total (*n* = 280)	CI (*n* = 211)	ICH (*n* = 69)	*p*-Value
**Type of impairment on rehabilitation admission (%)**				
Aphasia	52 (18.6)	37 (17.5)	15 (21.7)	0.476 ^c^
Dysphagia	140 (50.0)	101 (47.9)	39 (56.5)	0.267 ^c^
In-dwelling catheter	40 (14.3)	30 (14.2)	10 (14.5)	>0.950 ^c^
Incontinence	31 (11.1)	19 (9.0)	12 (17.4)	0.075 ^c^
Motor weakness	255 (91.1)	192 (91.0)	63 (91.3)	>0.950 ^c^
Sensory	112 (40.0)	74 (35.1)	38 (55.1)	0.004 ^c^
Nasogastric tube	47 (16.8)	25 (11.8)	22 (31.9)	<0.001 ^c^
**Cognitive impairment (%)**				
Yes (MOCA < 24)	196 (70.0)	148 (70.1)	48 (69.6)	>0.950 ^c^
No (MOCA ≥ 24)	84 (30.0)	63 (29.9)	21 (30.4)	
**Motor impairment**				
Upper-limb FMA, median (IQR)	37.0 (57)	43.0 (57)	26.0 (56)	0.080 ^a^
Lower-limb FMA, median (IQR)	21.5 (22)	22.0 (20)	14.0 (24)	0.064 ^a^
TIS, median (IQR)	13.0 (11)	14.0 (10)	11.0 (14)	0.014 ^a^

^a^ Mann–Whitney U test; ^c^ Fisher Exact test.

**Table 3:** Admission and discharge functional and impairment scores by stroke subtype.

Variable	Total (*n* = 280)	CI (*n* = 211)	ICH (*n* = 69)	*p*-Value
**Functional scores on admission**				
Total FIM, median (IQR)	72 (39)	74 (38)	67 (50)	0.006 ^a^
Motor FIM, median (IQR)	46 (30)	48 (29)	38 (33)	0.003 ^a^
Cognitive FIM, median (IQR)	26 (14)	27 (13)	24 (19)	0.079 ^a^
**Functional scores on discharge**				
Total FIM, median (IQR)	98 (41)	99 (37)	94 (42)	0.259 ^a^
Total FIM, 90 and below (%)	115 (41.1)	83 (39.3)	32(46.4)	
Total FIM, above 90 (%)	165 (58.9)	128 (60.7)	37 (53.6)	0.302 ^b^
Motor FIM, median (IQR)	69 (31)	71 (29)	68 (36)	0.316 ^a^
Cognitive FIM, median (IQR)	30 (11)	30 (11)	29 (11)	0.231 ^a^
Upper-limb FMA, median (IQR)	58 (54)	58 (53)	56 (54)	0.408 ^a^
Lower-limb FMA, median (IQR)	30 (14)	30 (12)	26 (21)	0.044 ^a^
TIS, median (IQR)	17 (8)	17 (8)	16 (10)	0.407 ^a^
**Calculated scores**				
FIM gain, median (IQR)	22 (18)	21 (15)	27 (23)	0.050 ^a^
FIM efficiency, median (IQR)	0.85 (0.73)	0.83 (0.70)	0.86 (0.90)	0.517 ^a^
UL FMA gain, median (IQR)	3 (10)	3 (9)	4 (16)	0.329 ^a^
LL FMA gain, median (IQR)	5 (8)	5 (8)	4 (7)	0.798 ^a^
TIS gain, median (IQR)	3 (5)	2 (5)	4 (7)	0.009 ^a^

^a^ Mann–Whitney U test, ^b^ Pearson Chi Square Test.

**Table 4:** Comparison of rehabilitation characteristics between two subgroups (n = 280).

Variable	Total (*n* = 280)	CI (*n* = 211)	ICH (*n* = 69)	*p*-Value
**Length of rehab stay**, median (IQR)	28.0 (24)	27.0 (20)	34.0 (32)	0.090 ^a^
**Medical complication at rehab**, *n* (%)				
Yes	110 (39.3)	84 (39.8)	26 (37.7)	0.778 ^b^
No	170 (60.7)	127 (60.2)	43 (62.3)	
**Discharge destination (%)**				
Home	245 (87.5)	187 (88.6)	58 (84.1)	0.401 ^b^
Institution/Others	35 (12.5)	24 (11.4)	11 (15.9)	
**Carer needed (%)**	230 (82.1)	170 (80.6)	60 (87.0)	0.279 ^b^

^a^ Mann–Whitney U test; ^b^ Pearson Chi Square test.

**Table 5:** FIM scores based on initial stroke severity, ICH based on GCS, and CI based on NIHSS.

**ICH Group**	**Mild (13–15)** ***n* = 45**	**Moderate-Severe (3–12)** ***n* = 24**	***p*-Value**
Admission FIM, median (IQR)	72.0 (39)	30.0 (33)	<0.001
Discharge FIM, median (IQR)	104.0 (31)	72.0 (43)	<0.001
FIM gain, median (IQR)	26.0 (19)	30.0 (36)	0.579
**CI Group**	**Mild (1–4)** ***n* = 82**	**Moderate-Severe (5–42)** ***n* = 121**	***p*-Value**
Admission FIM, median (IQR)	84.0 (23)	59.0 (35)	<0.001
Discharge FIM, median (IQR)	109.5 (23)	85.0 (40)	<0.001
FIM gain, median (IQR)	20.0 (17)	22.0 (16)	0.155

Mann–Whitney U test; 8 patients had missing NIHSS data. Notes: Gain refers to difference between scores recorded during admission to rehabilitation and at discharge.

**Table 6:** Multiple regression analyses for variables impacting total discharge FIM (N = 280).

Variables	Simple Linear Regression			Multiple Linear Regression		
	Coeff.	95% CI	*p*-Value	Adj. Coeff.	95% CI	*p*-Value
**Age**						
<65	Ref					
≥65	−7.988	−14.075, −1.901	0.010			
**Gender**						
Male	Ref					
Female	−8.515	−14.659, −2.372				
**Employment status**						
Unemployed	Ref			Ref		
Employed	13.814	7.855, 19.773	<0.001	6.012	2.801, 9.223	<0.001
**Stroke type**						
CI	Ref			Ref		
ICH	−4.800	−11.865, 2.264	0.182	4.781	1.043, 8.519	0.012
**Acute LOS**	−0.730	−0.937, −0.522	<0.001	−0.275	−0.399, −0.152	<0.001
**IDC**						
No	Ref					
Yes	−22.129	−30.457, 13.801	<0.001			
**NGT**						
No	Ref					
Yes	−27.741	−35.228, −20.254	<0.001			
**Tracheostomy**						
No	Ref					
Yes	−47.542	−69.911, −25.173	<0.001			
**VPS**						
No	Ref					
Yes	34.229	−16.809, 85.267	0.188			
**Complications**						
No	Ref					
Yes	−14.497	−20.512, −8.482	<0.001			
**Admission**						
TIS	2.702	2.412, 2.993	<0.001	0.707	0.355, 1.059	<0.001
Motor FIM	1.093	0.995, 1.191	<0.001	0.528	0.387, 0.670	<0.001
Cognitive FIM	2.078	1.857, 2.300	<0.001	0.865	0.635, 1.095	<0.001
UL FMA	0.529	0.429, 0.629	<0.001			
LL FMA	1.449	1.239, 1.660	<0.001			

The variable selection stepwise method was used; the adjusted R^2^ = 0.7591. R^2^ is the percentage of total variance explained by the model.

## Data Availability

The data presented in this study are available upon reasonable request from the corresponding author.

## References

[1] Roth G.A., Mensah G.A., Johnson C.O., Addolorato G., Ammirati E., Baddour L.M., Barengo N.C., Beaton A.Z., Benjamin E.J., Benziger C.P. (2020). Global Burden of Cardiovascular Diseases and Risk Factors, 1990–2019: Update from the GBD 2019 Study. J. Am. Coll. Cardiol..

[2] Ministry of Health Principal Causes of Death 2016. https://www.moh.gov.sg/resources-statistics/singapore-health-facts/principal-causes-of-death.

[3] Venketasubramanian N., Chen C.L. (2008). Burden of stroke in Singapore. Int. J. Stroke.

[4] Kong K.H., Yang S.Y. (2006). Health-related quality of life among chronic stroke survivors attending a rehabilitation clinic. Singap. Med. J..

[5] Teasell R. Chapter 5: The Efficacy of Stroke Rehabilitation. www.ebrsr.com.

[6] Cifu D.X., Stewart D.G. (1999). Factors affecting functional outcome after stroke: A critical review of rehabilitation interventions. Arch. Phys. Med. Rehabil..

[7] Chiu D., Peterson L., Elkind M.S., Rosand J., Gerber L.M., Silverstein M.D. (2010). Comparison of outcomes after intracerebral hemorrhage and ischemic stroke. J. Stroke Cerebrovasc. Dis..

[8] Jorgensen H.S., Nakayama H., Raaschou H.O., Olsen T.S. (1995). Intracerebral hemorrhage versus infarction: Stroke severity, risk factors, and prognosis. Ann. Neurol..

[9] Franke C.L., van Swieten J.C., Algra A., van Gijn J. (1992). Prognostic factors in patients with intracerebral haematoma. J. Neurol. Neurosurg. Psychiatry.

[10] Barber M., Roditi G., Stott D.J., Langhorne P. (2004). Poor outcome in primary intracerebral haemorrhage: Results of a matched comparison. Postgrad. Med. J..

[11] Paolucci S., Antonucci G., Grasso M.G., Bragoni M., Coiro P., De Angelis D., Fusco F.R., Morelli D., Venturiero V., Troisi E. (2003). Functional outcome of ischemic and hemorrhagic stroke patients after inpatient rehabilitation: A matched comparison. Stroke.

[12] Chae J., Zorowitz R.D., Johnston M.V. (1996). Functional outcome of hemorrhagic and nonhemorrhagic stroke patients after in-patient rehabilitation. Am. J. Phys. Med. Rehabil..

[13] Kelly P.J., Furie K.L., Shafqat S., Rallis N., Chang Y., Stein J. (2003). Functional recovery following rehabilitation after hemorrhagic and ischemic stroke. Arch. Phys. Med. Rehabil..

[14] Lipson D.M., Sangha H., Foley N.C., Bhogal S., Pohani G., Teasell R.W. (2005). Recovery from stroke: Differences between subtypes. Int. J. Rehabil. Res..

[15] Venketasubramanian N., Yoon B.W., Pandian J., Navarro J.C. (2017). Stroke Epidemiology in South, East, and South-East Asia: A Review. J. Stroke.

[16] Feigin V.L., Lawes C.M., Bennett D.A., Barker-Collo S.L., Parag V. (2009). Worldwide stroke incidence and early case fatality reported in 56 population-based studies: A systematic review. Lancet Neurol..

[17] Ng Y.S., Astrid S., De Silva D.A., Tan M.L.D., Tan Y.L., Chew E. (2013). Functional outcomes after inpatient rehabilitation in a prospective stroke cohort. Proc. Singap. Healthc..

[18] Chu C.L., Chen Y.P., Chen C.C., Chen C.K., Chang H.N., Chang C.H., Pei Y.C. (2020). Functional Recovery Patterns of Hemorrhagic and Ischemic Stroke Patients Under Post-Acute Care Rehabilitation Program. Neuropsychiatr. Dis. Treat..

[19] Verheyden G., Mertin J., Preger R., Kiekens C., Weerdt W.D. (2004). The Trunk Impairment Scale: A new tool to measure motor impairment of the trunk afterfckLRstroke. Clin. Rehabil..

[20] Fugl-Meyer A.R., Jääskö L., Leyman I., Olsson S., Steglind S. (1975). The post-stroke hemiplegic patient. 1. A method for evaluation of physical performance. Scand. J. Rehabil. Med..

[21] Beninato M., Gill-Body K.M., Salles S., Stark P.C., Black-Schaffer R.M., Stein J. (2006). Determination of the minimal clinically important difference in the FIM instrument in patients with stroke. Arch Phys Med Rehabil..

[22] Weimar C., Ziegler A., Konig I.R., Diener H.C. (2002). Predicting functional outcome and survival after acute ischemic stroke. J. Neurol..

[23] Salvadori E., Papi G., Insalata G., Rinnoci V., Donnini I., Martini M., Falsini C., Hakiki B., Romoli A., Barbato C. (2021). Comparison between Ischemic and Hemorrhagic Strokes in Functional Outcome at Discharge from an Intensive Rehabilitation Hospital. Diagnostics.

[24] Ministry of Health (2019). Singapore Stroke Registry Annual Report.

[25] Andersen K.K., Olsen T.S., Dehlendorff C., Kammersgaard L.P. (2009). Hemorrhagic and ischemic strokes compared: Stroke severity, mortality, and risk factors. Stroke.

[26] Deuschl G., Beghi E., Fazekas F., Varga T., Christoforidi K.A., Sipido E., Bassetti C.L., Vos T., Feigin V.L. (2020). The burden of neurological diseases in Europe: An analysis for the Global Burden of Disease Study 2017. Lancet Public Health.

[27] Ng Y.S., Jung H., Tay S.S., Bok C.W., Chiong Y., Lim P.A.C. (2007). Results from a prospective acute inpatient rehabilitation database: Clinical characteristics and functional outcomes using the Functional Independence Measure. Ann. Acad. Med..

